# A Review of Nutritional Regulation of Intestinal Butyrate Synthesis: Interactions Between Dietary Polysaccharides and Proteins

**DOI:** 10.3390/foods14213649

**Published:** 2025-10-26

**Authors:** Meiyu Yuan, Kaili Gao, Kaitao Peng, Shuang Bi, Xian Cui, Yuhuan Liu

**Affiliations:** 1 State Key Laboratory of Food Science and Resources, Engineering Research Center for Biomass Conversion, Ministry of Education, Nanchang University, Nanchang 330047, China; 2 College of Biological and Environmental Engineering, Jingdezhen University, Jingdezhen 334000, China; 3 School of Food and Health, Beijing Technology & Business University, Beijing 100048, China; 4 Chongqing Research Institute of Nanchang University, Chongqing 402660, China

**Keywords:** polysaccharide, protein, butyrate, gut microbiota, biodiversity

## Abstract

Butyrate is a key short-chain fatty acid (SCFA) essential for maintaining colon health, immune homeostasis, and metabolic balance. Its synthesis primarily depends on the fermentation of dietary substrates by the gut microbiota. In a healthy state, carbohydrate fermentation sustains the stability of the gut microbiota; however, in chronic diseases, the diversity of the microbiota decreases, and the metabolic pathway shifts from carbohydrate fermentation to protein fermentation, thereby inhibiting butyrate production. Polysaccharides and proteins play key roles in regulating butyrate synthesis. As fermentable carbon sources, polysaccharides promote the proliferation of probiotics, lower colonic pH, and inhibit anaerobic fermentation of proteins. However, excessive protein fermentation produces branched-chain fatty acid (BCFA), ammonia, phenols, and other metabolites that inhibit butyrate production. The structural characteristics of polysaccharides and the digestibility of proteins can determine the substrate selection of gut microbiota. This review systematically elucidates the biosynthetic pathways of butyrate in the gut and the host metabolic signaling pathways in which it participates, focusing on the interactions between dietary proteins and polysaccharides and their key regulatory mechanisms affecting butyrate production by the gut microbiota.

## 1. Introduction

At the intestinal level, butyrate exerts regulatory effects on metabolism, facilitates the transcellular transport of fluids, inhibits inflammatory processes, and induces the formation of epithelial defense barriers [1]. Butyrate is the most important SCFA for many physiological processes, including colon and liver. Butyrate naturally exists in the intestines and has a high concentration. Several studies have found that the ratio of acetate to propionate to butyrate in the colon of healthy individuals (regardless of region) has been found to be approximately 60:20:20 [2,3]. These ratios may vary with the individual, diet, microbial composition and health status. Acetate, propionate, and butyrate are all absorbed by the colonic mucosa; however, butyrate is preferentially transported and seems to be the preferred energy source for colonic epithelial cells [4]. In the complex microecosystem of the gut, microbial fermentation substrates are primarily derived from undigested dietary components, including proteins and polysaccharides [5,6]. As a key dietary constituent, polysaccharides selectively promote the growth of butyrate-producing bacteria such as *Faecalibacterium prausnitzii* and *Roseburia* spp. and modulate butyrate fermentation pathways [7,8,9]. However, under certain pathological conditions like inflammatory bowel disease and irritable bowel syndrome, gut microbiota composition and metabolic functions can be disrupted, leading to reduced butyrate production [5]. This is often accompanied by enhanced anaerobic protein metabolism, generating potentially harmful metabolites such as ammonia, amines, phenols, and hydrogen sulfide, which exhibit mucosal toxicity and may exacerbate disease progression.

Current research on dietary regulation of gut microbiota and metabolites has largely focused on single nutrients, providing fundamental insights into microbial metabolic principles [10]. Yet, in real dietary contexts, polysaccharides and proteins coexist and are simultaneously fermented by gut microorganisms. Their interactions remain complex and underexplored. Key research gaps include how the coexistence of polysaccharides and proteins influences microbial community structure and functional preference—specifically, the shift between glycolytic and proteolytic metabolism [11]. This metabolic orientation directly determines the efficiency of butyrate synthesis and the production of harmful metabolites such as ammonia and hydrogen sulfide. Furthermore, the structural characteristics of polysaccharides (e.g., glycosidic bonds and branching degree) combined with different protein sources (e.g., plant vs. animal protein) may exert synergistic or antagonistic effects on butyrate production. For example, low digestibility potato protein has been shown to reduce butyrate levels and increase protein hydrolysis metabolites, while certain proteins can promote short chain fatty acid synthesis [12,13]. Polysaccharide supplementation has been shown to reduce the concentration of harmful metabolites, while starch supplementation does not have this effect [14]. An appropriate polysaccharide-to-protein ratio appears crucial for maintaining gut microbial homeostasis and facilitating butyrate generation. Therefore, understanding how polysaccharide–protein interactions shape microbial metabolic pathways is vital for optimizing intestinal butyrate production and sustaining microbiota balance.

This review systematically examines the synthesis and signaling pathways of intestinal butyrate, with a particular focus on the comprehensive regulatory effects of dietary polysaccharides (especially molecular structural characteristics) and proteins on butyrate production. The article provides an in-depth analysis of how polysaccharides influence butyrate production by regulating the fermentation of colon proteins, and reveals that the interaction network of proteins and polysaccharides synergistically activate specific bacterial groups and optimize the microenvironment of butyrate synthesis through mechanisms such as cross-feeding. By combing the relevant research results, our objective is to establish a scientific foundation for future dietary intervention and gut microbiota treatment strategies.

## 2. Methods

### 2.1. Literature Search Strategy

To ensure a comprehensive and unbiased synthesis of the current knowledge, a systematic literature search was conducted. The primary electronic databases utilized for this review were PubMed, Scopus, and Web of Science. The search strategy was designed to capture all relevant studies investigating the interplay between dietary components and intestinal butyrate production. The search employed a combination of keywords. Core search terms included the following: Butyrate-related: “butyrate”, “butyric acid”, “butyrate synthesis”, “butyrate production”. Substrate-related: “dietary polysaccharide”, “dietary fiber”, “resistant starch”, “prebiotic”, “dietary protein”, “protein fermentation”, “amino acid”. Context-related: “gut microbiota”, “intestinal fermentation”, “colon”, “cecum”, “short-chain fatty acid”. The search was initially performed for articles published from January 1995 to December 2025. The reference lists of identified relevant reviews and primary articles were also manually screened to include any additional pertinent studies that might have been missed in the database search.

### 2.2. Eligibility Criteria

Inclusion Criteria: Original research articles (both in vivo animal studies and human clinical trials) and high-quality reviews that provided mechanistic insights. Studies that directly investigated the effect of dietary polysaccharides and/or proteins on intestinal butyrate concentration or synthesis. Studies that explored the synergistic, additive, or antagonistic interactions between dietary polysaccharides and proteins in regulating butyrate levels. Articles published in English.

Exclusion Criteria: Studies that only measured total short-chain fatty acids without specifically reporting butyrate data. Studies focused solely on in vitro fermentation without connection to a dietary intervention. Conference abstracts, editorials, and non-peer-reviewed publications. Studies where the full text was not accessible.

### 2.3. Data Extraction and Synthesis

The data selection process was conducted systematically to ensure objectivity and comprehensiveness. All records retrieved from the database searches were imported into EndNote X9 (Clarivate Analytics, Philadelphia, PA, USA) for management. The main inclusions were:

Study Characteristics: First author, publication year, and study type (e.g., rodent experiment, human trial, crossover design). Intervention Details: Type and dose of dietary polysaccharide and/or protein, duration of the intervention, and control diet. Model Information: Species, strain, and baseline characteristics of subjects (human or animal). Key Outcomes: Primary findings on butyrate concentration (in feces, cecal, or colonic contents), shifts in butyrate-producing microbiota, and changes in relevant metabolic pathways. Mechanistic Insights: Reported mechanisms, such as cross-feeding interactions between polysaccharide degraders and butyrate producers, or the impact of protein-derived metabolites (e.g., ammonia and BCFA) on butyrate synthesis.

Given the heterogeneity in experimental designs, substrates, and analytical methods across the studies, a narrative synthesis approach was adopted. The extracted data were systematically compared and organized into thematic sections to provide a coherent overview of the current evidence and to identify consensus views, controversies, and knowledge gaps in the field.

## 3. Synthesis and Metabolic Pathways of Butyrate

### 3.1. Synthesis of Butyrate from Polysaccharides and Proteins

**Figure 1:**
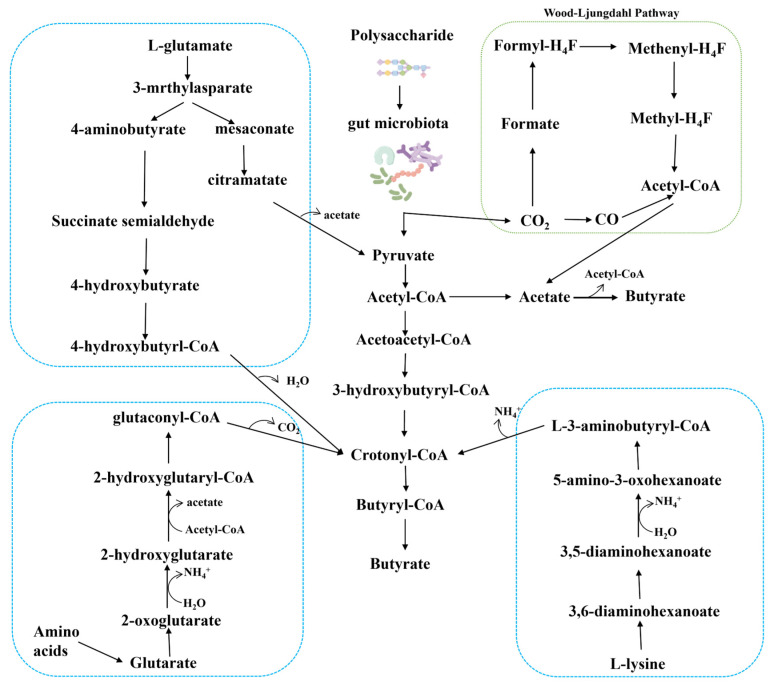
This figure illustrates the relevant pathways involved in the fermentation of AAs, such as glutamate and lysine, as well as the fermentation of polysaccharides to produce butyrate within the metabolism of gut microbiota. It integrates the Wood–Ljungdahl pathway, highlighting the material transformations and interactions among various metabolic routes. Different colored dashed boxes represent distinct metabolic pathways: the blue dashed box encompasses amino acid metabolism pathways; the green dashed box indicates the Wood–Ljungdahl pathway; and the central section depicts the fermentation of polysaccharides by gut microbiota to produce butyrate.

The production of butyrate in the gut primarily utilizes pyruvate, glutarate, 4-aminobutyrate, and lysine as substrates. The synthesis of butyrate from pyruvate follows the pathway illustrated in Figure 1. During polysaccharide fermentation, dietary carbohydrates are degraded into monosaccharides, which are converted through glycolysis into pyruvate and then into acetyl-CoA, a key intermediate for butyrate synthesis. Acetate, formed from pyruvate or via the Wood–Ljungdahl pathway, also serves as a major carbon source for butyrate [15]. Finally, butyrate is produced from acetyl-CoA through a series of condensation and reduction steps catalyzed by butyryl-CoA transferases [16,17,18].

Butyrate can also be produced through the fermentation of peptides and amino acids (AAs), but this pathway is uncommon in the metabolism of gut microbiota in healthy populations. It is estimated that the number of amino acid fermenting bacteria accounts for less than 1% of the gut microbiota [19]. Butyrate is the main fermentation product of glutamic acid, lysine, histidine, cysteine, serine, and methionine. It is synthesized from glutamate via the 3-methylaspartate pathway (*Clostridium* spp.) or crotonyl-CoA (4-aminobutyrate pathway, and 2-hydroxyglutarate pathway). These pathways are predominantly found in the *Firmicutes* phylum, including *Acidaminococcus fermentans*, *Clostridium sporophaeroides*, *Clostridium symbiosum, Fusobacterium* spp., and *Pepperstreptococcus asaccharolyticus*.

### 3.2. Absorption and Signal Transmission of Butyrate

Colonic epithelial cells metabolize butyrate through β-oxidation and the tricarboxylic acid cycle. While small intestinal cells can also absorb butyrate, they primarily rely on glucose and glutamate for energy [20]. Butyrate crosses the colonic cell membrane either by diffusion or via specific transporter proteins, mainly including the monocarboxylate transporter protein (MCT) and the sodium-conjugated monocarboxylate transporter protein (SMCT). MCT is a transporter protein in the solute carrier family [21], and its working mechanism is to couple a proton (H^+^) to transport butyrate into cells in an electrically neutral manner. Among the more than ten known MCT subtypes, four (MCT1, MCT2, MCT3, and MCT4) have been confirmed to have the ability to transport SCFA. Butyrate is primarily absorbed through MCT1 [22], which is located on the apical membrane of colonic epithelial cells, whereas MCT4 is specifically expressed in the basolateral membrane. SMCT is a specific transporter protein, SMCT1 and SMCT2 are expressed only in the parietal membrane and help to transport butyrate across the cell membrane by coupling with sodium ions. The functional role of MCT1 in butyrate transport was demonstrated through quantitative uptake assays using [^14^C]-labeled butyrate in the intestinal epithelial cell line [23]. Through heterologous expression in Xenopus oocytes, Seiji Miyauchi and colleagues demonstrated that SLC5A8 (SMCT1) functions as a Na^+^-coupled transporter for SCFA [24]. In addition to the aforementioned butyrate uptake system, breast cancer resistance protein (BCRP) is involved in the removal of butyrate from cells. In normal colonic epithelial cells, butyrate is absorbed by MCT1 and SMCT1 as the main energy source. Unmetabolized butyrate is excreted by BCRP, thereby reducing its intracellular concentration [25].

Histones are proteins that wrap DNA within the nucleus. Together with DNA, they form nucleosomes, the basic structural units of chromatin. Chemical modifications on histones—especially the acetylation of lysine residues in their tails—serve as key epigenetic switches that regulate gene expression. Acetylation of histones introduces negatively charged acetyl groups, neutralizing the positive charge and loosening their tight binding to DNA, thereby forming an open euchromatin structure. This structural shift allows key transcriptional regulatory elements to access and bind target DNA sequences, directly activating gene transcription initiation. Conversely, histone deacetylases (HDACs) catalyze the removal of acetyl groups, promoting chromatin condensation into a dense, closed heterochromatin state. This effectively hinders the access and binding of transcription elements to DNA, resulting in gene repression of expression. At the level of gene expression regulation, butyrate is a classic HDAC inhibitor that increases the acetylation level of histone H3 and H4, change the chromatin conformation, and facilitates transcriptional activation of genes. The molecular mechanism involves butyrate specifically inhibiting the enzymatic activity of class I and class IIa HDACs, leading to histone hyperacetylation in the nucleus. Through the primary cholangitis model, it has been proven that butyrate inhibited HDACs function, leading to enhanced acetylation of lysine 27 on histone H3 at promoter regions of PPARD and FAO genes in myeloid-derived suppressor cells (MDSCs) [26]. Importantly, the HDAC inhibitory effect of butyrate is highly context-dependent.

**Figure 2:**
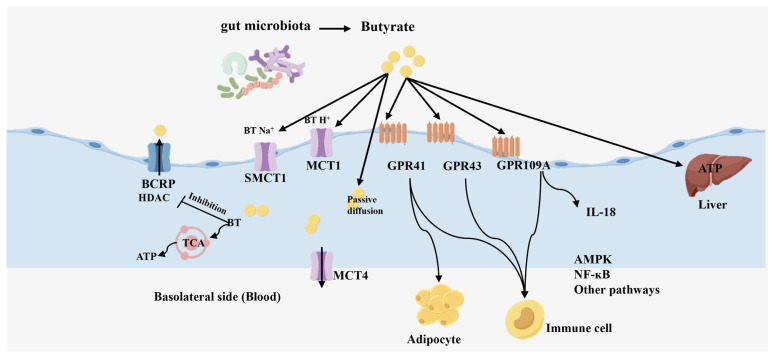
Schematic diagram of intestinal absorption and transport mechanism of butyrate. This figure shows that butyrate, produced by intestinal microbial fermentation, is absorbed by colonic epithelial cells through apical membrane transporters (MCT1, SMCT1). It is primarily utilized as an energy substrate to generate ATP via β-oxidation. Butyrate functions as a signaling molecule to activate G protein-coupled receptors (GPR41, GPR43, GPR109A). This activation regulates the release of anti-inflammatory factors (IL-18), energy sensing pathways (AMPK), and inflammatory responses (NF-κB), ultimately coordinating local immune cell function and distal organ metabolism.

In terms of signaling, butyrate acts through three G protein-coupled receptors (GPR43 (FFAR2), GPR41 (FFAR3), and GPR109A (HCAR2) [27] (Figure 2). Wang et al. used cryo-electron microscopy technology combined with functional experiments such as site directed mutagenesis to elucidate at the atomic level how SCFA are recognized and bound by receptors, and ultimately trigger the entire process of G protein coupled receptors [28]. These receptors show specificity for different SCFA. For instance, butyrate exhibits a preferential binding affinity for GPR41, whereas GPR43 has a higher affinity for acetate and propionate [29]. GPR43 is most highly expressed in immune cells, including monocytes and neutrophils [15,16,21]. GPR41 is present in adipose tissue and immune cells. It has been found that GPR41 mainly transmits signals by activating G protein of Gαi/o family. In contrast, GPR43 was able to activate G proteins of the Gαi, Gαq and Gα12 families. In terms of binding affinity for endogenous ligands, the order of potency for GPR41 is: propionate = valerate = butyrate > acetate. While the relative ligand potency order of GPR43 is: propionate = butyrate = acetate [30]. In the colonic lumen, butyrate is produced by the gut microbiota at high concentrations (10–20 mM) and acts as an endogenous agonist of GPR109A.

GPR109A (HCAR2) plays a crucial role not only in immune regulation but also in maintaining homeostasis and immune responses in intestinal epithelial cells. When butyrate activates GPR109A on colonocytes, it triggers intracellular signaling cascades, promotes the secretion of the anti-inflammatory cytokine IL-18, and enhances autophagy, collectively contributing to intestinal stability [31]. In immune cells, butyrate inhibits the maturation of dendritic cells (DCs) and macrophages, reducing their antigen-presenting capacity and secretion of pro-inflammatory cytokines, thereby preventing the overactivation of T cells. Additionally, butyrate induces the expansion of regulatory T (Treg) cells and inhibits the differentiation of Th17 cells, promoting the production of anti-inflammatory factors such as IL-10 and TGF-β, thus facilitating systemic immune tolerance. Melissa D. et al. demonstrated that GPR109A activation by its endogenous ligand butyrate is a key mechanism for reducing inflammation, as shown by transplanting GPR109A/toxemia cells into a mouse graft-versus-host disease (GVHD) model [32]. Lucija Leko et al. compare disease severity between GPR109A knockout mice and wild-type mice revealing that butyrate exerts its anti-inflammatory effects by activating GPR109A receptors, thereby inhibiting IL-1 β and its downstream IL-23/IL-17A inflammatory axis [33]. Moreover, butyrate reduces the level of reactive oxygen species by activating the Nrf2 antioxidant pathway and enhancing glutathione (GSH) synthesis, and alleviate stress damage to the to intestinal barrier and immune cells. Butyrate also enhances epithelial barrier function by upregulating the expression of tight junction proteins such as Claudin-1, Occludin, and ZO-1 in intestinal epithelial cells. It plays a crucial role in repairing and strengthening the intestinal barrier by downregulating the transcription of pro-inflammatory genes, including IL-1β, IL-6, and COX-2, thereby inhibiting the inflammatory cascade. Wang et al. also found that butyrate depressed the protein abundance of myosin light chain kinase (MLCK), elevated the expression of tight Junction (TJ) proteins, and restored the cellular distribution of TJ proteins and the barrier function of epithelial cells [34]. Simultaneously, the GPR109A signaling pathway enhances cellular autophagy, facilitating the clearance of damaged organelles and misfolded proteins. This process helps cells maintain metabolic homeostasis and respond effectively to stress. Additionally, it acts on goblet cells in the upper intestinal mucosa, strongly stimulating them to produce and secrete mucus. This mucus effectively isolates numerous symbiotic bacteria and potential pathogens in the intestine, preventing direct contact with epithelial cells and thereby avoiding unnecessary immune activation.

## 4. The Effect of Dietary Components on Butyrate

The fermentation of easily digestible carbohydrates usually does not produce large amounts of butyrate. These easily digestible carbohydrates include simple sugars (such as glucose and fructose) and easily digestible starches found in staple foods like rice and noodles. They are rapidly broken down by digestive enzymes in the small intestine, providing insufficient substrate for the gut microbiota to ferment and generate substantial butyrate. In contrast, the fermentation of indigestible carbohydrates produces butyrate, such as oligosaccharides, resistant starch, cellulose, hemicellulose (Xylan), and pectin [35]. Numerous studies have shown that the intake of non-digestible carbohydrates (NDCs) can influence butyrate production. For example, in vivo experiments have demonstrated that consuming polysaccharides leads to a sustained increase in butyrate concentration within the cecum of rats. Conversely, protein fermentation in the intestine plays a key factor driving the overall structural alterations of the microbial community, and butyrate is one of the fermentation products derived from peptide and amino acid fermentation during this process [19]. The fermentation of protein serves as an energy source, resulting in the production of organic acids and the accumulation of other deleterious end products [36]. Individuals who adopted a low carbohydrate and high protein diet pattern exhibited an increase in harmful metabolites, including BCFA and N-nitroso compounds, while the content of butyrate decreases, compared to those maintaining body weight with a high protein intake. In the human colon, there appears to be a significant tradeoff between glycolysis and protein hydrolysis fermentation and it largely depends on diet [37]. This indicates that the quantity of fermentable carbohydrates, rather than the meat in the diet, is the crucial factor for butyrate producers.

### 4.1. The Effect of Polysaccharides on Butyrate

NDCs are key substrates utilized by butyrate-producing bacteria in the gut to produce butyrate and is influenced by the properties of NDC. The upper gastrointestinal tract lacks enzymes capable of degrading complex polysaccharides, allowing these compounds to reach the colon intact, where they serve as fermentation substrates for the colonic microbiota. Butyrate-producing bacteria, such as *Faecalibacterium prausnitzii* and *Roseburia* spp., secrete specific glycoside hydrolases (GHs) through polysaccharide utilization loci (PULs) encoded in their genomes to hydrolyze polysaccharides, producing assimilable oligosaccharides and monosaccharides [38]. These products undergo glycolysis to form pyruvate, which is ultimately converted into butyrate via enzymes such as butyryl-CoA and acetyl-CoA transferase. Table 1 shows the effects of various types of NDCs on butyrate production and its associated bacteria, as well as the role of NDCs in promoting butyrate production. Different types of NDCs exhibit significant variations in acid production. The molecular structure of NDCs profoundly influences their impact on the gut microbiota. Some researchers believe that oligosaccharides have relatively simple structures and do not require complex primary degradation. They can be quickly recognized and directly utilized by gut microorganisms, leading to rapid fermentation in the proximal colon and producing a significant peak in SCFA concentration. For example, Yuan and Yu et al. demonstrated that oligosaccharides are more effective than polysaccharides in producing SCFA [5,39]. Oligosaccharides derived from polysaccharides increased the concentration of SCFA in fecal samples. However, most researchers believe that the complex structure of polysaccharides enables them to resist rapid fermentation in the proximal colon. They serve as “escaping fermentation substrates” that reach the distal colon, ensuring a continuous and stable supply of SCFA throughout the entire colon [40,41]. Similarly, polysaccharides from different sources produce varying amounts of SCFA. The seemingly contradictory viewpoints mentioned above reveal fundamental differences in the fermentation patterns of different carbohydrates. The root cause lies in the fact that molecular weight (Mw), types of glycosidic bonds, and monosaccharide composition together constitute their “structural complexity.” These factors synergistically determine the rate, location, and ultimate metabolic fate of their utilization by microorganisms. Therefore, it is not credible to broadly compare the functions of the two major categories—considering specific structural features. Consequently, the impact of structural characteristics on butyrate production has been systematically discussed.

#### Effects of Different Polysaccharide Structures on Butyrate

Mw: The Mw of polysaccharides is a key structural parameter that determines their utilization efficiency by gut microbiota, thereby regulating the composition of the gut microbiota and the production of SCFAs, especially butyrate. Polysaccharides with different Mw ranges can selectively enrich specific microbial populations and differentially influence metabolic outcomes. For example, compared to other polysaccharides, low-Mw polysaccharides derived from medicinal fungi and blackberry polysaccharides are more easily utilized by the fecal microbiota, resulting in favorable alterations in the microbiota. Generally speaking, the production rate of the SCFAs of low Mw polysaccharides is faster, but this does not affect the final SCFA yield [42]. Guar gum with Mw in the range of 10–15 kDa primarily promotes the production of butyrate during fermentation, whereas other Mw ranges tend to produce acetic acid [43]. At the microbial community level, different microbial populations exhibit distinct preferences for the Mw of polysaccharides. This Mw-dependent utilization is evident across bacterial taxa: high-Mw polysaccharides (>100 kDa) are preferentially degraded by butyrogenic families such as *Ruminococcaceae,* whereas taxa like *Actinobacteria* and *Prevotellaceae* exhibit limited capacity to utilize these complex substrates. Conversely, medium-Mw polysaccharides show significant correlations with the enrichment of *Prevotellaceae*, *Bacilli*, and *Coprococcus*, all of which contribute to butyrate through various metabolic pathways. In contrast, low-Mw polysaccharides often promote the proliferation of bacterial communities such as *Proteobacteria*, *Actinomycetes*, and *Enterobacteriaceae*, which are associated primarily with acetate rather than butyrate as the main fermentation end-product [44]. The Mw of polysaccharides profoundly influences butyrate synthesis levels by modulating the ecological fitness and metabolic preferences of the gut microbiota. This structure-function relationship provides a mechanistic basis for the targeted design of dietary polysaccharides to steer microbial community function and improve host health outcomes.

Glycosidic bond: Studies have shown that *Clostridium butyricum* can effectively break down β (1-4)- glycosidic bonds. Therefore, guar gum, mannooligosaccharides and galactooligosaccharides can significantly enhance the abundance of *Clostridium butyricum* [45]. The β (1-4) glycosidic bonds may contribute to the enhancement of butyrate production by oligosaccharides and play a similar role in butyrate-producing bacteria. In addition, elevated starch solubility also significantly promotes the production of SCFAs. Fructooligosaccharides containing β (1-2) and α (1-2) glycosidic bonds also promote the increase in butyrate production [46]. *Bifidobacterium* and *Lactococcus* indirectly promote butyrate production through “cross-feeding” by providing precursors such as lactic acid, while *Lachnospiraceae* is the primary contributor to the direct synthesis of butyrate [47]. Studies have shown that during inulin fermentation, the abundance of *Bifidobacterium* increases significantly and is positively correlated with the presence of the 1,2-Fru*p* structure. Oligogalactose containing 1,6-Gal*p* exhibits stronger growth-promoting effects on *Bifidobacterium* than oligogalactose containing 1,4-Gal*p* [48]. T-Glcp was significantly positively correlated with the relative abundance of *Butyricimonas*, while 1,4-Gal*p* was significantly associated with the enrichment of the *Lactococcus* and the *Lachnospiraceae NK4A136* [49]. Both 1,3-Fuc*p* and 1,4-Fuc*p* linked to increases in similar butyrate-producing bacteria, including *Allobaculum, Lachnospiraceae NK4A136, Akkermansia,* and *Faecalibacterium*) [44]. Specifically, distinct glycosidic bonds selectively enrich butyrate-producing bacteria that possess the corresponding enzymatic capabilities. β(1-4) glycosidic bonds serve as key targets for *Clostridium butyricum*; consequently, polysaccharides rich in these bonds—such as guar gum and mannooligosaccharides—effectively promote its proliferation [50]. Structures like 1,2-Fru*p* and 1,6-Gal*p* have been shown to significantly and positively correlate with the enrichment of *Bifidobacterium*, while T-Glc*p* and 1,4-Fuc*p* are closely associated with the abundance of other butyrate-producing bacteria, such as *Butyricimonas* and *Lachnospiraceae NK4A136.* During the fermentation of polysaccharides linked by different glycosidic bonds, bacteria responsible for butyrate synthesis in the gut microbiota may undergo specific enrichment. Therefore, the types of glycosidic bonds in polysaccharides can indirectly affect the final efficiency of butyrate synthesis by regulating microbial composition and function.

Monosaccharide composition: Monosaccharide composition, as the fundamental unit of polysaccharides, directly determines the utilization efficiency and metabolic pathways of specific gut microbiota, thereby systematically regulating butyrate synthesis. Different types of monosaccharides significantly influence the efficiency of butyrate production due to their distinct chemical properties and microbial utilization mechanisms. Numerous studies have shown that certain monosaccharides exhibit stronger butyrate-promoting effects, and their mechanisms are closely related to the metabolic preferences of specific microbial communities. Experimental evidence reveals a significant increase in cecal and distal colonic butyrate production with lactulose (galactose and fructose) supplementation compared to lactitol (galactose and glucitol), highlighting the critical influence of monosaccharide configuration on the gut microbiota [51,52]. Xylose has been demonstrated to exert a more pronounced influence on butyrate production than glucose and glucuronic acid [11]. The internal mechanism involves specific butyrate-producing bacteria exhibiting high selectivity for monosaccharide substrates. For example, *Roseburia inulinivorans* can utilize glucose and fucose, whereas *Coprococcus* catus specializes in fructose fermentation [53]. Additionally, moderate levels of arabinose are significantly and positively correlated with the abundance of the butyrate-associated bacterium *Lachnospiraceae NK4A136.* In addition, the chain length of polysaccharides also significantly affects the generation of butyrate. Ji et al. demonstrated that the production and proportion of SCFA during the fermentation of inulin-type fructan (ITF) were influenced by chain length. As the ITF chain length increases, the molar ratio of butyrate decreases [54].

Consequently, the contribution of polysaccharide structure (Mw, glycosidic bonds, monosaccharides, chain length) to butyrate production is multifaceted and context-dependent, with no single factor emerging as universally dominant. Future research must therefore shift toward integrated, multi-omics approaches that can elucidate the precise structure–function–metabolism relationships within an individual’s gut ecosystem, enabling personalized nutritional strategies.

### 4.2. The Effect of Protein on Butyrate

Protein is considered a crucial factor influencing the fermentation process. There are two main sources of protein in the colon: endogenous proteins, including glycoproteins, mucins, and proteins produced in some pancreatic diseases. The increase in these proteins may be related to inflammation and ulceration; The second are exogenous proteins, which derived from dietary. These proteins are partially resistant to digestion in the stomach and small intestine, and therefore are not absorbed, depending largely on the total amount of protein ingested. Studies have shown that by regulating the protein content in the substrate, it may effectively affect the production of butyrate in intestinal microbial fermentation [55]. Butyrate can be produced from glutamate and lysine by *Clostridium* and *Megasphaeras.* This pathway involves the production of ammonia and is activated in the absence of carbon sources, which differs from the pyruvate pathway. However, in contrast to the pyruvate to butyrate pathway used by *Clostridium*, which involves the production of ammonia and is only activated in the absence of a carbon source. Therefore, it is essential to consider the optimal characteristics of proteins, including concentration, type, source, and fermentation rate, to avoid the production of harmful metabolites such as BCFA and ammonia. Table 2 lists the effects of different proteins and their intake on butyrate.

**Figure 3:**
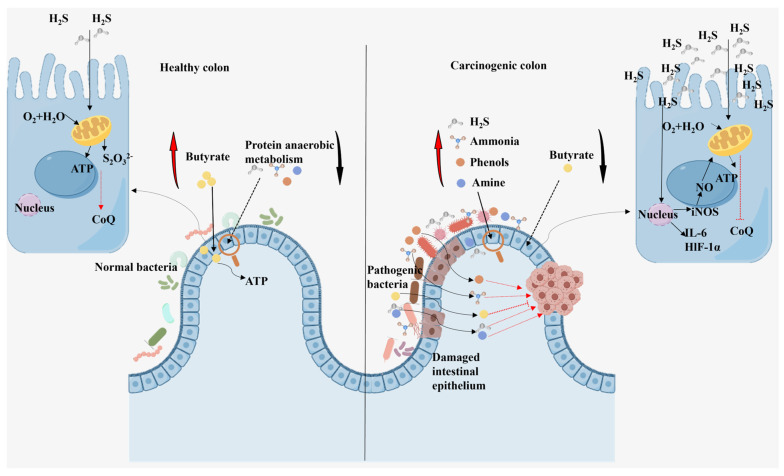
The effects of butyrate and amino acid catabolites in healthy population and colon cancer population. Compared with protein anaerobic fermentation, butyrate fermentation in healthy individuals is the predominant process, exerting a beneficial influence on the host. In colon cancer patients, the butyrate fermentation pathway is absent or impaired, and protein anaerobic fermentation is dominant, which promotes cancer progression. The effect of H_2_S on colonocytes: When the concentration of H_2_S is low, mitochondria are effectively oxidized to thiosulfate (S_2_O_3_^2−^), and oxygen consumption and ATP production are increased. When the concentration of H_2_S is too high and exceeds the detoxification capacity of the cell, cytochrome C oxidase activity is inhibited, resulting in a decrease in oxygen consumption and ATP production. High concentrations of intracytoplasmic H_2_S induces the expression of inflammation-related genes. Meanwhile, nitric oxide (NO) impairs the detoxification process of H_2_S and may exacerbate its harmful effects.

Protein critically influences the production and physiological effects of butyrate through two primary, interconnected mechanisms: (1) direct alteration of the gut microbial ecosystem, and (2) modulation of host metabolism Via specific fermentation metabolites. The relationship between protein intake and butyrate-producing bacteria is complex and non-linear. While proteins provide essential nitrogen for microbial growth, including that of butyrate producers, excessive intake—particularly of highly fermentable animal proteins—often leads to a decrease in the abundance of key butyrate-producing bacteria such as *Clostridium cluster XIVa*. This occurs because high-protein environments favor the proliferation of proteolytic bacteria (e.g., *Pseudomonas*, *Bacillus*) over saccharolytic taxa (e.g., *Bacteroidetes*, *Actinobacteria*) [55]. The resulting shift in community structure redirects metabolic flux away from carbohydrate fermentation toward protein catabolism. Additionally, when dietary protein intake is excessive, even highly digestible animal protein may exceed the processing capacity of the digestive system, thereby increasing protein spoilage in the colon [56]. Importantly, the metabolic consequences of this shift extend beyond the mere reduction in butyrate synthesis. Harmful metabolites generated during protein spoilage, such as ammonia and hydrogen sulfide (H_2_S), directly impair colonocyte function. Butyrate serves as the primary energy source for colonic epithelial cells, undergoing β-oxidation in their mitochondria. Both ammonia and H_2_S have been shown to inhibit this critical oxidative process [57]. This inhibition creates an energetic deficit in colonocytes, compromising barrier function and increasing epithelial apoptosis (Figure 3). Therefore, the detrimental effects of high-protein diets may stem not only from reduced butyrate production but also from a functional blockade of its utilization by the host.

This mechanistic understanding highlights the importance of optimizing protein characteristics—including source, concentration, and fermentation rate—to support a microbial community that sustainably produces butyrate while minimizing the generation of metabolites that impair its critical role in colonocyte health.

#### Regulation of Protein Digestibility on Butyrate Fermentation Metabolism

Protein digestibility is a crucial factor determining the amount of protein that reaches the colon, thereby influencing microbial fermentation processes and the subsequent production of metabolites such as butyrate. Plant proteins generally exhibit lower digestibility due to structural characteristics and the presence of antinutritional factors. Consequently, for a given intake level, a larger proportion of plant protein escapes digestion in the upper gastrointestinal tract and enters the colon, serving as a substrate for microbial fermentation. Initially, one might hypothesize that this increased colonic protein fermentation would be universally detrimental, given the association of protein-derived metabolites such as ammonia, phenols, and sulfides with mucosal irritation and carcinogenicity. Some studies have also reported adverse effects of plant protein. For instance, Jia et al. reported that plant protein could affect the gut microbiota via bile acid and lipid metabolism, leading to lipid deposition and liver damage [58]. However, epidemiological evidence contradicts this, showing that diets high in plant protein are associated with beneficial health outcomes [59,60,61]. Beyond its direct health benefits, emerging research indicates that parental intake of plant protein can offer protective metabolic effects for their offspring. Conversely, a paternal diet rich in animal protein appears to program an increased susceptibility to metabolic disorders in the next generation [62]. This paradox emphasizes that the impact on health depends on the amount of fermented protein, fermentation quality, and individual differences, and fermentation quality is inherently linked to protein sources. The critical mechanistic link lies in how different proteins modulate the gut microbial community and its metabolic pathways. Animal proteins, rich in sulfur-containing AAs, preferentially fuel bacteria that produce hydrogen sulfide and other potentially detrimental metabolites. In contrast, certain “resistant” plant proteins may selectively enrich for microbial taxa that channel fermentation towards more beneficial outcomes. This could occur through several mechanisms: (1) the amino acid profile of plant proteins may be less conducive to the generation of toxic metabolites; (2) plant proteins are often embedded in a food matrix rich in fiber, which can promote carbohydrate fermentation over protein fermentation, leading to increased butyrate production that improves gut barrier function and mitigates the effects of protein-derived toxins; and (3) specific plant proteins may act as prebiotics, directly stimulating butyrate-producing bacteria such as *Roseburia* and *Faecalibacterium.*

The digestion of proteins is influenced by their structure, which determines how they are broken down. The primary structure of a protein dictates the specific sites where enzymes cleave the peptide bonds. Meanwhile, the secondary, tertiary, and quaternary structure of the protein further influences the ability of proteolytic enzymes to successfully access the core backbone of the protein, which in turn affects overall digestive efficiency [63]. For example, the increase in β-sheet content promotes the formation of dense, structured protein aggregates, which reduces protein digestibility [64]. Additionally, the type and quantity of bonds also affect the protein structure, consequently affecting digestibility [65]. The presence of disulfide bonds in rapeseed protein serves to enhance its stability and hinders protein digestion in the human body [66]. Additionally, antinutritional factors, protein aggregation, and reactions with crosslinking agents can affect protein digestibility. For example, AAs deficiencies in legumes (sulfur-containing AAs) and grains (lysine), as well as antinutritional factors such as protease inhibitors, polyphenols, phytates, hemagglutinin, fiber, and non-starch polysaccharides, can interfere with protein digestion and absorption [66]. However, it is important to note that processing plant proteins can enhance their digestibility and significantly reduce antinutritional factors. This reduction decreases the amount of undigested protein reaching the colon, thereby lowering proteolytic activity in that region. Additionally, dietary composition plays a crucial role in modulating the microbial response to undigested proteins.Therefore, the association between a protein source and butyrate levels is not a simple function of digestibility, but a complex consequence of how the protein shapes the entire gut ecosystem. The current challenge is to move beyond correlative observations to a mechanistic understanding of how specific dietary proteins influence microbial composition and function to ultimately affect host health.

### 4.3. Polysaccharide-Mediated Regulation of Colonic Protein Metabolism and Its Impact on Butyrate Production

**Figure 4:**
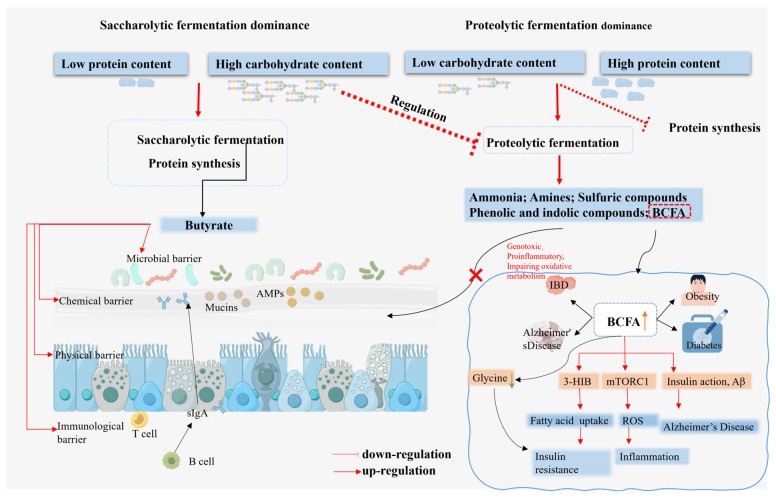
The balance between saccharolytic and proteolytic fermentation as influenced by the ratio of carbohydrates to protein, and its impact on gut health and disease progression. A high proportion of carbohydrates helps regulate the fermentation of protein and butyrate, thus exerting a beneficial influence on intestinal health, including increasing the expression of antimicrobial peptides and mucins, inhibiting the growth of harmful bacteria, and reducing the expression of pro-inflammatory factors. In contrast, high protein intake is dominated by protein fermentation, which produces harmful metabolites, such as BCFA. Elevated BCFA levels are associated with diseases such as obesity, diabetes, and Alzheimer’s disease. The catabolic intermediate of valine, 3-HIB, promotes the accumulation of lipids in muscle, thereby triggering insulin resistance. Furthermore, elevated BCFA concentrations have been linked to reduced glycine levels, with this alteration exhibiting a positive correlation with insulin resistance. High levels of BCFA can also stimulate the mTOR signaling pathway, which can subsequently induce oxidative stress and inflammatory responses. Furthermore, it can also lead to impaired hypothalamic insulin signaling, and exacerbate the hyperphosphorylation of Tau in Alzheimer’s disease (AD) patients. Antimicrobial peptide (AMP); Secretory immunoglobulin A (SIgA); 3-hydroxyisobutyric acid (3-HIB); microtubule-associated protein Tau (Tau).

When dietary polysaccharide intake is insufficient, the metabolic patterns of intestinal microorganisms undergo significant changes. The metabolic activity of proteolytic bacteria, which utilize proteins and AAs as their primary substrates, is enhanced. These bacterial groups produce a series of potentially harmful metabolites [67]. These substances not only exert direct toxicity on intestinal epithelial cells by disrupting TJ but can also penetrate the intestinal wall and enter the circulatory system, increasing the risk of systemic inflammation and metabolic diseases (Figure 4). The availability of polysaccharide can affect protein fermentation for three main reasons. The fermentation of polysaccharides produces high concentrations of SCFA, resulting in a significant decrease in pH in the proximal colon [68]. This acidic environment exceeds the optimal pH range for most proteases and peptidases, thereby slowing the degradation of dietary proteins and peptides. Additionally, specific protein fermentation pathways are inhibited in the presence of polysaccharides. The transcription of genes related to amino acid catabolism, such as deaminase genes, is suppressed, resulting in a reduction in the amino acid deamination process. Finally, polysaccharides promote the proliferation of glycolytic bacteria, such as *Bifidobacterium* and *Lactobacillus* [69,70]. During their rapid growth, these bacteria assimilate large amounts of nitrogen sources—primarily AAs and ammonia—from the environment into proteins and nucleic acids. This process competitively consumes the amino acid substrates available to proteolytic bacteria and shifts metabolism from energy-producing catabolism toward biosynthesis, thereby systematically reducing the production of protein fermentation byproducts.

Therefore, polysaccharide intake not only alters the composition and function of the intestinal microbial community but also mitigates the adverse effects of a high-protein diet on proteolytic metabolism. Studies have shown that polysaccharide intervention can significantly reduce the production of intestinal protein fermentation products, with urinary excretion of p-cresol serving as an effective biomarker of proteolytic activity. After ingestion of arabinoxylan oligosaccharides (AXOS), a significant reduction in urinary excretion of p-cresol was observed (more than 50%) [71]. In contrast, starch supplementation did not cause this effect [14]. These results confirm that proteolysis becomes more intense in the distal colon, and polysaccharide with distal fermentation potential can effectively regulate the metabolic activity of protein hydrolyzing bacteria.

### 4.4. Activation of Microbiota Through Cross Feeding Promotes Butyrate Production: Synergistic Effect of Polysaccharides and Protein

**Figure 5:**
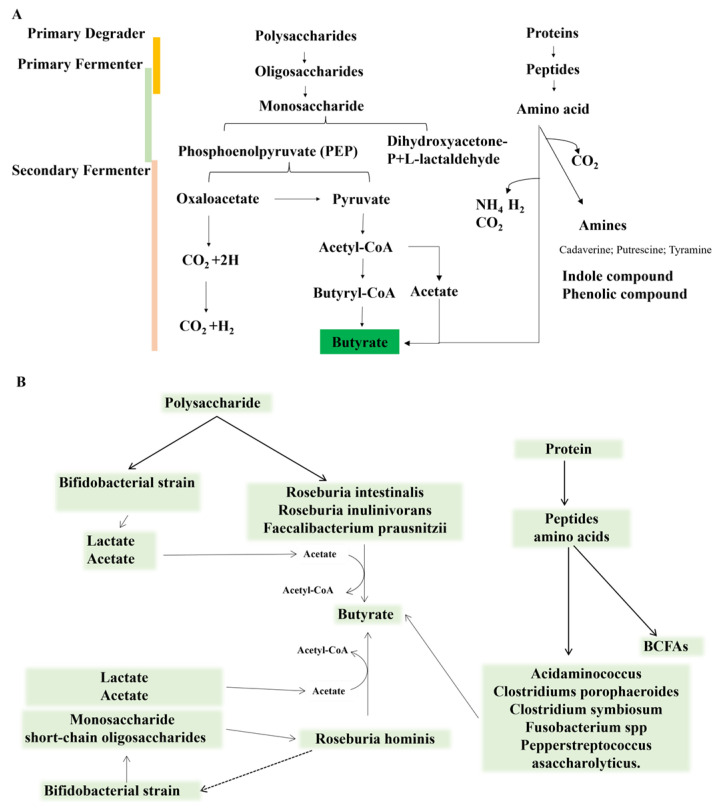
(**A**) Described the transformation process of butyrate production from polysaccharide and protein fermentation: primary degraders, secondary fermenter, and secondary f fermenter. (**B**) Partial gut microbiota involved in the cross-feeding fermentation of polysaccharides and proteins to produce butyrate.

After entering the colon, polysaccharides serve as fermentation substrates for gut microbiota and are broken down into butyrate. Specific microorganisms and metabolic pathways involved may include the following (Figure 5): (1) Polysaccharide can serve as a direct source of nutrition and energy for certain butyrate-producing bacteria, enabling them to produce butyrate. (2) Selective fermentation of polysaccharide: Upon reaching the intestine, polysaccharides are fermented by specific beneficial bacteria, such as *Bifidobacteria*. The superior carbohydrate metabolism of *Bifidobacterium* promotes intestinal colonization of the genus and also benefits other intestinal symbionts, especially butyrate-producing bacteria, through cooperative metabolism [72]. (3) Cross-feeding is the foundation of butyrate production in the human colon. Many glycoside hydrolases with diverse catalytic specificities have been identified. However, no single bacterial species encodes all the enzymes required to break down every type of polymeric carbohydrate. Some bacteria ferment polysaccharide to produce intermediate metabolites, which are utilized by another class of bacteria and further fermented to produce butyrate [73]. Primary degraders: in the process of cross-feeding, primary degraders (such as *Bacteroides* and *Bifidobacterium*) employ a multitude of carbohydrate enzymes to decompose polysaccharide to generate products of primary fermentation, including oligosaccharides, monosaccharides, acetate, lactate, CO_2_ and H_2_ [74,75]. These primary metabolites promote the growth and metabolism of certain bacteria collectively referred to as secondary degraders [76]. Secondary degraders are generally divided into four categories: acetogens, sulfate-reducing bacteria, methanogens and butyrate-producing bacteria. *Faecalibacterium prausnitzii*, *Anaerostipes caccae* and *Ruminococcus* produce butyrate as an end product [17]. There are two main types of cross-feeding: One is that bacteria utilize oligosaccharides or monosaccharides released from prebiotic substrate to produce butyrate through fermentation or other metabolic pathways, which is a mechanism unrelated to the utilization of lactate and acetate utilization. The other is that bacteria consume the primary metabolites produced by polysaccharide fermentation, such as acetate and lactate, thus providing precursors for the synthesis of butyrate. These two cross-feeding interactions facilitate the production of butyrate in the gut. Using *Bifidobacteria* as an example, cross-feeding by *Bifidobacteria* can enhance the ecological adaptation of butyrate-producing bacteria [77]. In general, selective fermentation and cross-feeding of *Bifidobacteria* will occur simultaneously. It is evident that *Bifidobacteria* do not produce butyrate because the pathway responsible for its production is absent from their genome. Nevertheless, yet the bifidogenic effect is usually accompanied by a butyrate effect [78]. Type 1 cross-feeding interactions occur between *Bifidobacteria* that degrade polysaccharide and butyrate-producing bacteria that are unable to degrade polysaccharide. Type 2 cross-feeding interaction occurs between *Bifidobacteria* capable of degrading oligosaccharides and butyrate-producing bacteria that rely on the acetate pathway, etc. [79].

In gut microbial communities, AAs act as a multifunctional currency, fueling central metabolism energy production, supplying nitrogen for transamination, and serving as essential nutrients for auxotrophs. This creates a nitrogen-centric food web, distinct from the *Bacteroidales*-driven carbon web. This nitrogen network is propelled by various *Firmicutes* (e.g., *Clostridium*, *Actinomyces*), which metabolize AAs though pathways like the Stickland reaction, enabling cross-feeding through the exchange of AAs and ammonium (NH_4_^+^). In the colon, NH_4_^+^ is an abundant nitrogen source produced by microbes, with its concentration increasing distally. Beyond NH_4_^+^, AAs represent a second major form of nitrogen exchange during cross-feeding. Amino acid auxotrophs are present in virtually all microbial communities, providing an ecological basis for widespread amino acid cross-feeding. The prevalence of this cooperation stems from its energetic efficiency: rather than incurring the high metabolic cost of de novo synthesis, microorganisms can directly utilize “ready-made” AAs released by other strains in the environment for their anabolic needs [80,81]. The entry of dietary protein into the colon serves as the primary source of these nitrogenous compounds, and its digestibility not only determines the substrate supply but also fundamentally shapes the metabolic output of the gut microbiota. Feeding protein well digested in the small intestine such as casein may result in the imbalance of the ratio between carbohydrates and protein as fermentative substrates in the large intestine while feeding resistant protein may correct this imbalance and promote SCFA production, butyrate in particular [82,83]. Similarly, the metabolites produced by protein fermentation can alter the nutritional environment of the colon and regulate the metabolic activity of microorganisms. Notably, the fermentation of certain AAs, such as glutamic acid and lysine, can generate butyrate precursors, including acetyl-CoA, which directly participate in the butyrate synthesis pathway. Gases such as hydrogen and CO_2_ produced by protein degradation can serve as substrates for methanogens and sulfate-reducing bacteria. These bacterial groups engage in metabolic interactions with butyrate-producing bacteria, forming a complex cross-feeding network (Figure 5). Therefore, proteins and polysaccharides have complementary effects in providing carbon and nitrogen sources, and their synergistic fermentation optimizes the metabolic efficiency of microbial communities. By maintaining appropriate protein and polysaccharide intake, the growth and metabolism of butyrate-producing bacteria can be promoted, ultimately enhancing colonic butyrate production.

**Table 1:** The effects of various types of NDCs on butyrate.

Type of Carbohydrate	Research Contents	Butyrate Concentration	Butyrate-Related Bacteria	Butyrate-Related Role	Reference
Oat β-glucan	Mice (food allergy model)	restored butyrate to normal level	*Lachnospiraceae* ↑	improves inflammation and colon damage	[50]
sea buckthorn polysaccharide	ICR mice	increasing by 4.52 times	*Agathobactere, Lachnospiraceae_NK4A136_group*, *Lachnospiraceae*, *Alistipes*	-	[84]
Resistant starch	db/db mice (diabetes model)	The concentrations of butyrate significantly increased	-	reduces intestinal permeability and enhances immune health of the kidney in diabetes	[85,86]
high amylose maize butyrylated starch (HAMSB)	Sprague–Dawley rats	10% HAMSB: 200.11 µmol > Control: 17.98 µmol	-	the greater ability of HAMSB to deliver butyrate to the large bowel and lots of butyrate	[87]
Safflower Dietary Fiber	Constipation Rats	18.17–27.25 mM/g	*Clostridium*, *Ruminococcaceae* ↑	intestinal peristalsis and barrier repair	[88]
Barley arabinoxylan	Mice (hyperglycemia)	Butyrate concentration ↑	*Bifidobacterium* ↑	affect L-cell differentiation, GLP-1 ↑	[89]
*Codonopsis pilosula* neutral polysaccharide	Immunocompromised mice	regressed and exceeded the control group	*Roseburia*, *Eubacteriu, Clostridium* ↑	the strongest correlation with immune activation	[8]
*Astragalus* polysaccharide	Immunosuppressed broilers	4.36 µmol/g, 1.5-fold higher than control group	*Ruminococcaceae UCG-014*, *Oscillibacter*, *Shuttleworthia*	improve the growth performance of immunosuppressive broilers	[90]
Pectin	Tumor-bearing mice humanized	reversed the decrease in butyrate	*Lactobacillus*, *Roseburia*, *Faecalibacterium*	the function of CD4+ T cells, the IFN-γ on CD8+ T cells and the efficacy of anti-PD-1 mAb ↑	[91]
Inulin	C57BL/6J mice (alcoholic liver disease model)	2-fold higher than AF/CON group	-	promoted M1 Mψs and inhibit M2 Mψs, reduced inflammation	[92]
pectin and inulin (1:1)	Induction of collagen-induced arthritis (CIA) mice	2.5–4 times higher than ND group	*Bifidobacterium*	reducing joint inflammation and the immune-inflammatory response mediated by CD4 T cells	[93]
Fructooligosaccharide	Elderly PlyFermS fermentation model	stimulated butyrate formation (+116%–123%)	*Lachnospiraceae, Bifidobacterium* spp., *Roseburia* spp.	metabolic activity of elderly colonic microbiota ↑	[94]
Fructooligosaccharide	Fecal	7.15 ± 2.28 mM	*Clostridium cluster IX*	-	[95]
Hydrolysed guar gum	Colitis mice	stimulated the production of butyrate	*Clostridium cluster XIVa*	prevents the development of TNBS-induced colitis	[96]
Glucomannan Oligosaccharide	C57BL/6 mice (obesity model)	<25 mM/g	*Ruminococcaceae* ↑	gene Muc2, acetylated histone proteins H3 and H4 ↑	[97]
Alginate oligosaccharide	intestinal mucositis	About 13.63 mM/g	*Lachnoclostridium*, *Muribaculaceae*	modulate TLR4/MyD88/NF-κB pathway, anti-inflammatory, regulate cytokine levels	[98]
Mannan oligosaccharide	5xFAD mice (Alzheimer’s disease mouse model)	Enriched the serum and fecal butyrate levels	*Clostridium_pasteurianum*, *Lachnospira*	alleviated the cognitive decline and Aβ accumulation	[99]

Note: HCD (high-cholesterol diet), alcohol-fed (AF) group (AF/CON), Mψs (macrophages), normal diet (ND), Anti-human PD-1 monoclonal antibody (anti-PD-1 mAb), Glucagon-Like Peptide-1 (GLP-1), Interferon-gamma (IFN-γ), ↑ indicates an up-regulation.

**Table 2:** The effects of different proteins and their intake on butyrate.

Type of Protein	Research Objects	Protein Concentration/ Ratio	Butyrate/Butyrate-Producing Bacteria	Effect	Reference
Soy protein β-conglycinin (β-CG)	Mice (heart failure model)	20% β-CG	Butyrate, *Butyricimonas,* *Anaerotruncus* ↑	ameliorated TAC-induced LV remodeling	[100]
crude protein (CP)	Pigs	Low-CP group:16% protein vs. normal-CP group: 18% protein	butyrate in the jejunum and total SCFA in the ileum, *Clostridium cluster IV, XIVa, Bifidobacterium* ↑	pro-inflammatory cytokines levels (IL-8, IFN-γ and TNF-α), ammonia ↓, up-regulated the expression of jejunal ZO-1, and ileal MUC2 and OCLN	[101]
Protein	Sprague-Dawley mice	Low protein	*Enterobacteriaceae,* the concentration of butyrate ↑	a low-protein diet could modulate the microbial ecology in the large intestine of neonatal rats.	[102]
Protein	Birds	Low protein	butyrate ↑	intestinal integrity and immune functions ↑	[103]
casein or β-CG	Mice	20% protein	*Butyricimonas*, butyrate ↑	-	[12]
potato protein (PP) or casein	Sprague-Dawley mice	containing casein, or PP at 250 g/kg diet for 10 d	The content of butyrate in PP group was higher than that in casein group	casein: predominant acid was succinate. PP: predominant acid was butyrate.	[104]
CP	Sprague-Dawley mice	50% CP group vs. 14% CP group	50% CP: pro-inflammatory flora: *Collinsella*, acid ↑ vs. 14% protein: the abundance of SCFA producing-bacteria ↑	50% CP group: the concentration of serum urea nitrogen, liver injury indicators (ALT and AST) ↑. 14% CP diet improved colonic microbial amino acid metabolism.	[105]
dietary CP	newly weaned pigs	20%, 17.16%, 15.30%. 13.90% CP	as CP level decreased, butyrate, *Clostridium cluster XIVa* and *Lactobacillus* ↑	As CP level decreased, ammonia concentration ↓, large intestinal bacterial community ↑.	[106]
Diet	Obese but otherwise healthy male	29% high protein	butyrate decreased in concentration (by 50%), *Roseburia, Eubacterium rectale, Lachnospiraceae* ↓	BCFA, isovalerate and isobutyrate ↑, carcinogenic NOCs ↑.	[107]
Casein (CAS), soy protein (SOY)	healthy male and female	CAS: 34% energy SOY: 31% energy	Feces and urine butyrate ↓	BCFA, 2-methylbutyrate ↑.	[108]
Soy protein	8-week-old male mice (high-fat diet)	23.78% soy protein VS 22.86% casein Diet	Butyrate, *Enterococcus*, and *Ruminococcus* were significantly higher in the Soy than Casein group	reduced serum cholesterol and fatty acid synthesis related genes expression levels.	[109]
Milk protein	Colitis mice	53% protein	*Clostridium XIVa*, *Faecalibacterium*, *Roseburia genera* ↓	The concentration of H_2_S and colitis severity ↑.	[110,111]
Beef protein	mice (high-fat diet)	-	the abundance of butyrate-producing bacteria such as *Anaerotruncus*, *Butyricicoccus*, and *Lactobacillus* in HFB ↓	enhanced cumulative energy intake, increased level of LDL-C, TC, and TG concentration in serum	[112]

TAC (transverse aortic constriction), LV (left ventricular), CP (crude protein), HFS (high-fat diet + soy protein), HFB (high-fat diet + beef protein), Alanine Aminotransferase (ALT), Aspartate Aminotransferase (AST), Casein (CAS), soy protein (SOY), N-Nitroso Compounds (NOCs), Low-Density Lipoprotein Cholesterol (LDL-C), Total Cholesterol (TC), Triglycerides (TG), Interleukin-8 (IL-8), Interferon-gamma (IFN-γ), Tumor Necrosis Factor-alpha (TNF-α), Zonula Occludens-1 (ZO-1), Mucin 2 (MUC2), Occludin (OCLN), ↑ indicates an up-regulation, ↓ indicates an down-regulation.

Under the premise of sufficient carbon sources (energy), butyrate-producing bacteria tend to prioritize AAs for biomass synthesis and bacterial proliferation, rather than converting them into putrefactive metabolites through fermentation pathways. When both carbon and nitrogen sources are sufficiently supplied, the abundance of butyrate-producing bacteria increases, enabling more efficient conversion of intermediate metabolites such as acetate into butyrate. Therefore, proteins and polysaccharides have complementary effects in providing carbon and nitrogen sources, and their synergistic fermentation optimizes the metabolic efficiency of microbial communities. By maintaining appropriate protein and polysaccharide intake, the growth and metabolism of butyrate producing bacteria can be promoted, ultimately enhancing the production of colonic butyrate.

## 5. Conclusions

Butyrate production by gut microbiota is a complex metabolic process, which is deeply affected by the synergistic interaction between dietary polysaccharides and proteins. The selection of metabolic pathways of these two substrates by flora directly affects the synthesis efficiency of butyrate. Under sufficient supply of polysaccharide, butyrate-producing bacteria can preferentially utilize nitrogen sources, such as AAs, for synthesizing cellular components and proliferation, rather than for generating harmful protein fermentation products. When sufficient carbon and nitrogen sources are present in the microbial environment, the number and metabolic activity of butyrate–producing bacteria increase significantly, thereby more efficiently converting precursor substances such as acetate into butyrate. Therefore, maintaining an appropriate ratio of protein to polysaccharide in the diet can help promote the enrichment and metabolic activity of butyrate-producing bacteria, ultimately enhancing the biosynthesis level of butyrate in the colon.

A central challenge in current research on gut microbiota and butyrate production lies in determining the optimal dietary ratio of polysaccharides to proteins. Although existing evidence underscores the importance of this ratio in influencing butyrate synthesis, it remains impossible to establish a universal recommendation due to significant inter-individual variability in gut microbiota composition and the scarcity of systematic human intervention studies. Current understanding still relies heavily on in vitro and animal models, while the relationship between polysaccharide structure and fermentability remains insufficiently elucidated. Moreover, findings regarding the impact of proteins—particularly plant-based proteins—on butyrate production are inconsistent, and the underlying mechanisms are poorly characterized.

To address these gaps, future research should focus on the following directions: (1) Determining the optimal dietary polysaccharide-to-protein ratio to maximize butyrate synthesis in the human body through dose–response studies. (2) Utilizing multi-omics technologies to accurately analyze the microbially mediated network and quantify the metabolic flux from protein and polysaccharide to butyrate synthesis. (3) Investigate the relationship between polysaccharide structure and its biological activity. (4) Examine how variations in gut microbiota composition among individuals influence their responses to combined polysaccharide-protein intervention strategies.

## Data Availability

The original contributions presented in the study are included in the article, further inquiries can be directed to the corresponding authors.
